# Cardiac tamponade – a rare cause of sudden death in autoimmune polyglandular syndrome

**DOI:** 10.1007/s12024-024-00851-2

**Published:** 2024-06-18

**Authors:** Luzern Tan, John D. Gilbert, Roger W. Byard

**Affiliations:** 1 Adelaide Medical School, The University of Adelaide, Level 2, Room N237, Frome Road, Adelaide, South Australia 5005 Australia; 2 Forensic Science South Australia, 21 Divett Place, Adelaide, South Australia 5000 Australia

**Keywords:** Autoimmune polyglandular syndrome type 2, Addison disease, Autoimmune hypothyroidism, Fibrinous pericarditis, Tamponade

## Abstract

A 27-year-old male died suddenly due to cardiac tamponade arising from pericarditis complicating autoimmune polyglandular syndrome (APS) type 2. He had a history of primary Addison disease and autoimmune hypothyroidism which were corroborated at autopsy. In addition a florid fibrinous pericarditis was associated with 287 g of turbid fluid in the pericardial sac. Although pericarditis with tamponade is a potential complication of APS, it has rarely if ever, been reported as a cause of sudden death. Lethal mechanisms may involve both compressive and restrictive effects.

## Case report

A 27-year-old male who had complained of chest pains for two days was found unresponsive in bed. Resuscitation was attempted but was unsuccessful. His relevant past history included a diagnosis of Addison disease at the age of 15, with a hospital admission five years prior to death with an Addisonian crisis. In the previous year raised thyroid stimulating hormone (TSH) levels suggestive of hypothyroidism were found, however the decedent was reported to have been reluctant to commence treatment and ceased medical follow up.

**Fig. 1:**
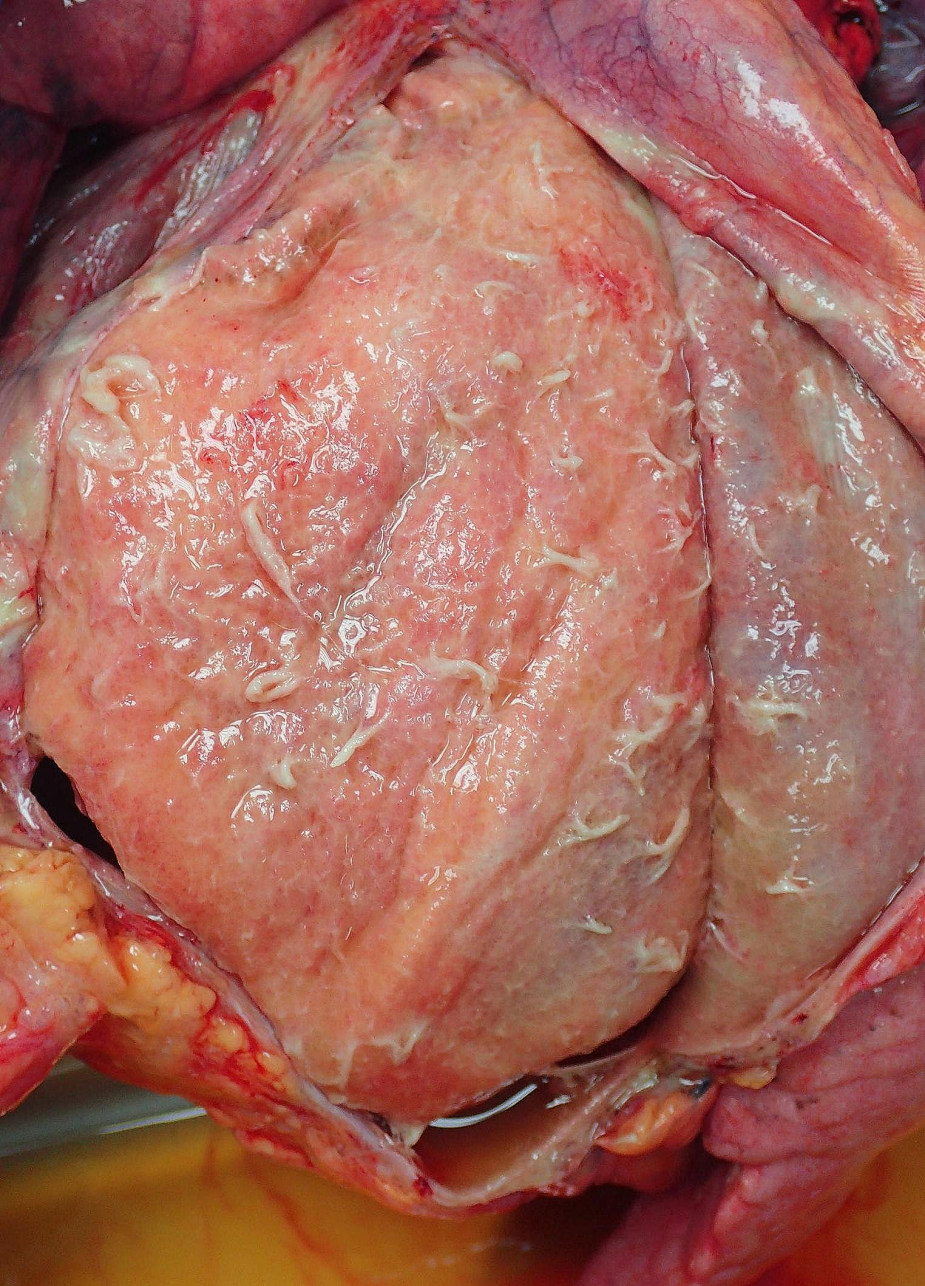
A florid fibrinous pericarditis in a 27-year-old man with APS type 2–287 g of free fluid were drained from the pericardial sac

At autopsy the major findings were in the pericardial cavity where a florid fibrinous pericarditis was associated with 287 g of turbid fluid (Fig. 1). The heart was of normal weight (459 g) and structure with levocardia, atrial situs solitus and atrioventricular and ventriculoarterial concordance. The cardiac chambers were of normal size and mural thickness. They were opened in a standard fashion commencing in the right atrium and auricle and extending through the tricuspid valve into the right ventricle. The foramen ovale was closed The pulmonary outflow tract was unremarkable. Opening of the left atrium and auricle revealed no abnormalities. The left ventricle was unremarkable with a normal aortic outflow tract. Cardiac valves were all of normal configuration and dimension with no evidence of endocarditis. The cut surfaces of the myocardium were normal with no evidence of myocarditis or fibrosis. The venous return to the heart was normal. Coronary artery ostia were normally situated and widely patent. The coronary arteries were right dominant with no atherosclerotic material and were normal aside from myocardial bridging of the mid portion of the left anterior descending artery (over a distance of 3 cm and up to 0.5 cm in depth). Bilateral pleural effusions (each containing 400mL of slightly turbid, serous fluid) along with pulmonary edema and congestion were noted. Other significant findings were marked pallor of the thyroid gland and atrophy of the adrenal glands. Analysis of peripheral blood showed no alcohol and non-toxic concentrations of paracetamol (~ 21 mg/L) and codeine (~ 0.024 mg/L).

**Fig. 2:**
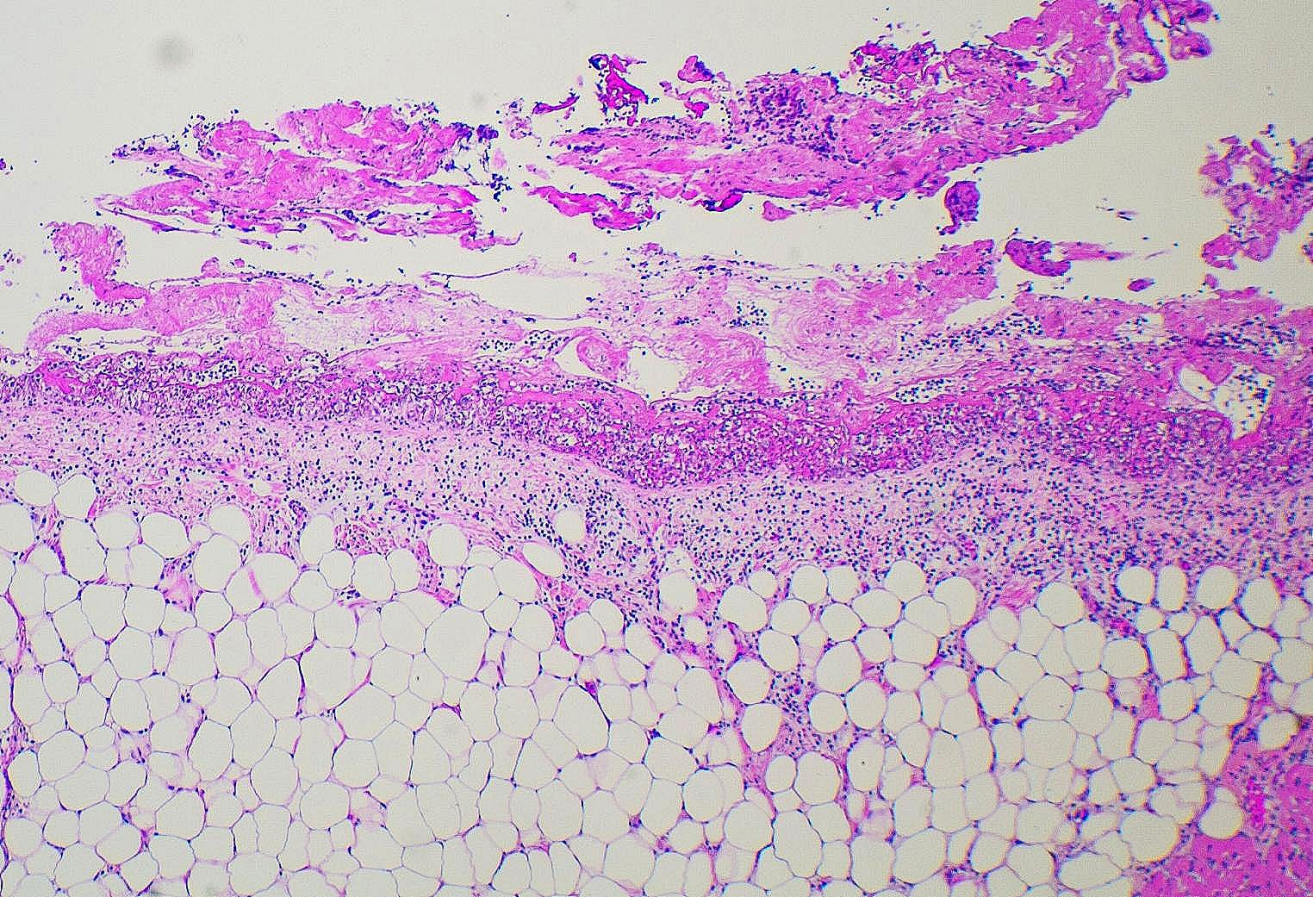
Histologic examination of the pericardial sac showed a fibrinous exudate containing patchy infiltrates of neutrophils, with plump reactive mesothelial cells (Hematoxylin and eosin, H&E x 150)

**Fig. 3:**
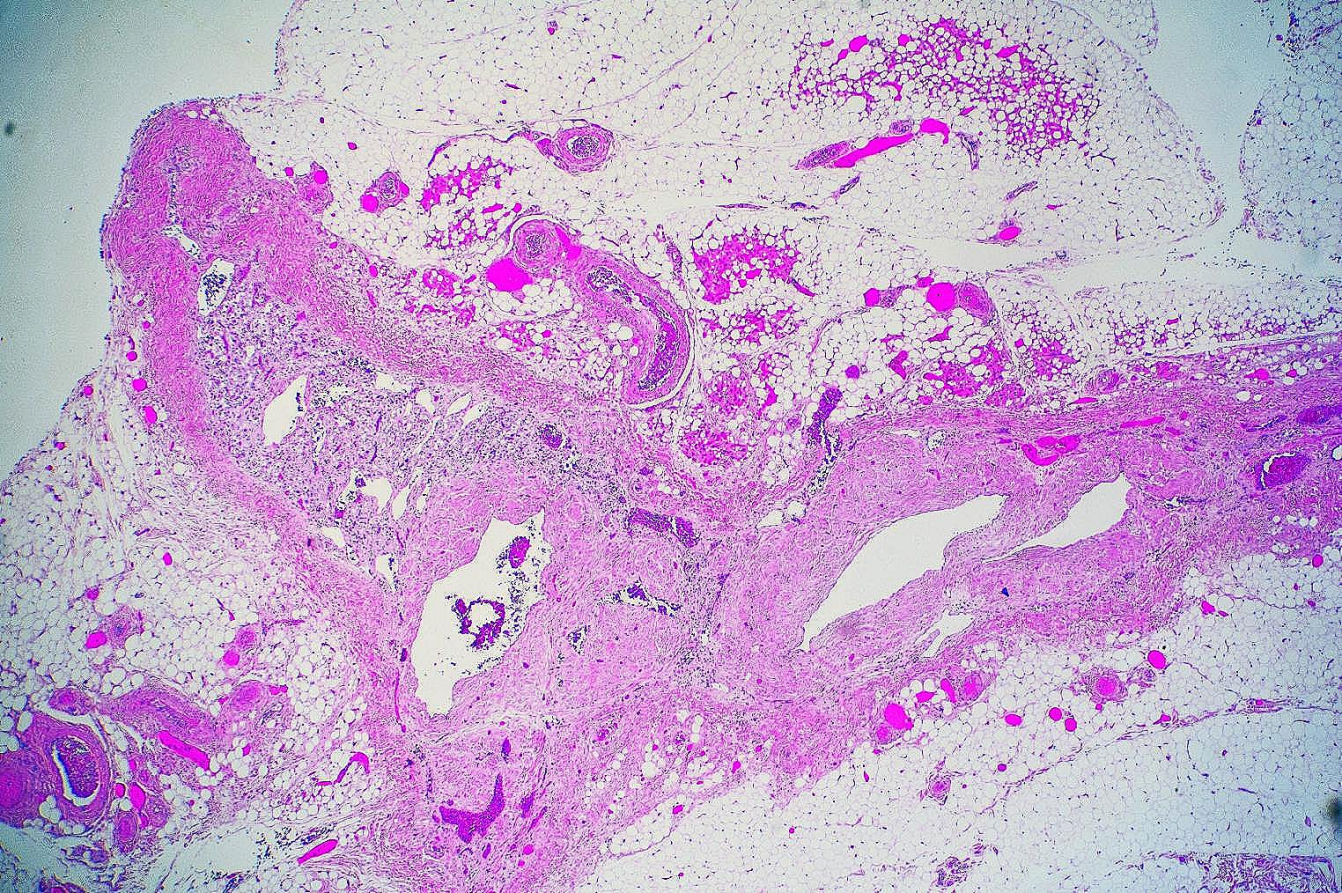
Histological examination of the adrenal gland showed marked atrophy and fibrosis in keeping with Addison disease (H&E x 150)

**Fig. 4:**
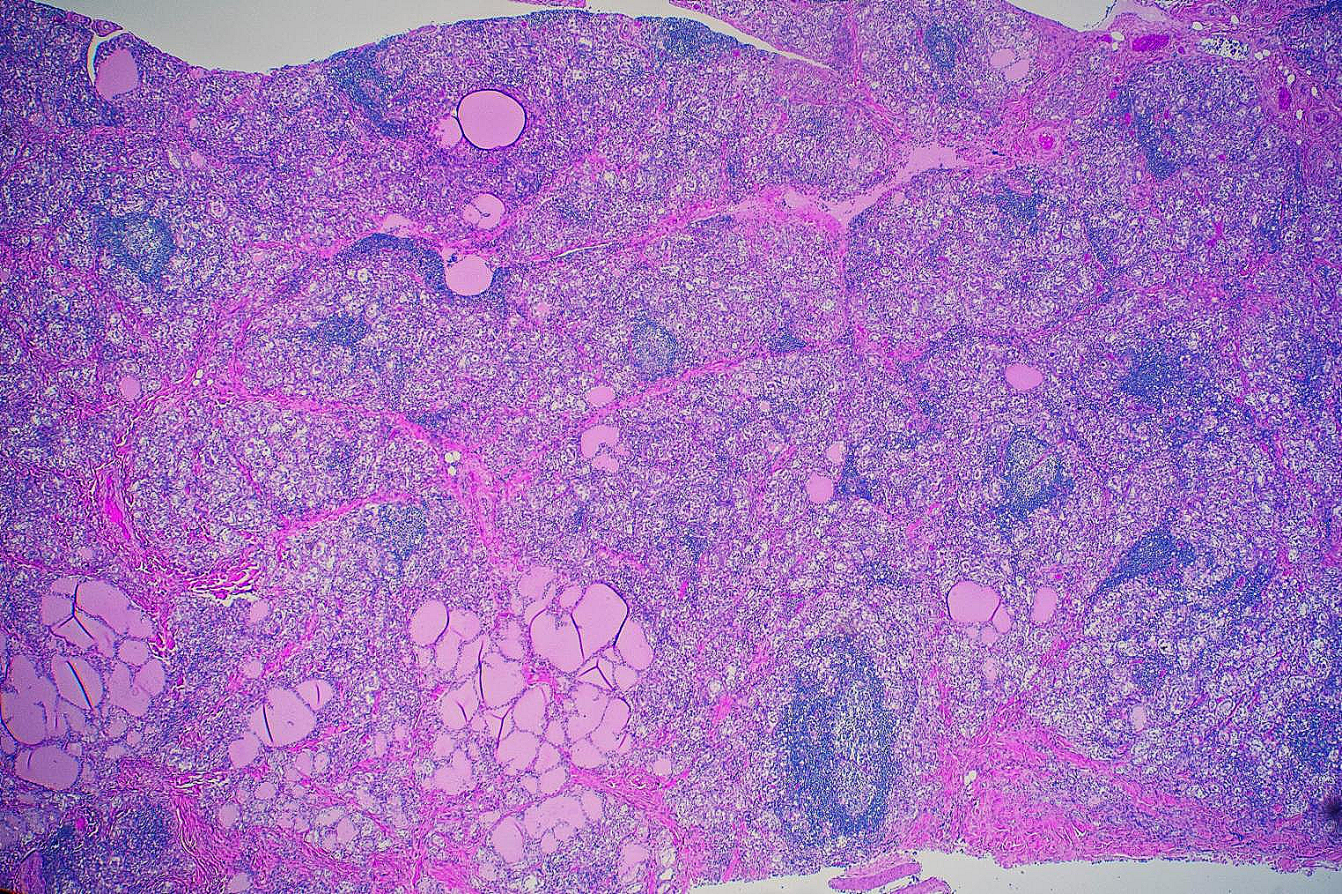
Histological examination of the thyroid gland showed a florid lymphocytic thyroiditis with prominent germinal centre formation (H&E x 150)

Histology of the myocardium showed no evidence of myocardtis with coating of the epicardial surfaces by a fibrinous exudate containing patchy infiltrates of neutrophils, with plump reactive mesothelial cells (Fig. 2). The adrenal glands showed marked atrophy and fibrosis (Fig. 3) in keeping with Addison disease. The thyroid gland showed a florid lymphocytic thyroiditis with prominent germinal centre formation (Fig. 4). Bacterial culture performed on the pericardial fluid showed no growth and no viruses were detected. Biochemical analysis of vitreous humor showed evidence of mild renal impairment with hyponatremia. Glucose and β-hydroxybutyrate levels were normal. There were no other underlying organic diseases present which could have caused or contributed to death and there was no evidence of trauma.

In keeping with the decedent’s history and autopsy findings, autoimmune polyglandular syndrome (APS) type 2 was diagnosed and death was attributed to cardiac tamponade due to complicating pericarditis.

## Discussion

APS is an autoimmune condition that affects multiple endocrine organs concurrently resulting in progressive impairment of function over an often insidious course [1]. It was first described in 1926 by Schmidt, a German pathologist, in a patient with hypothyroidism and adrenal insufficiency with biopsies showing lymphocytic infiltration of the thyroid and adrenal glands [2]. In the reported case typical florid lymphocytic infiltration of the thyroid gland was present (Fig. 4).

APS has been divided into three types: APS type 1, APS type 2 and X-linked immunodysregulation, polyendocrinopathy, and enteropathy (IPEX), the features of which are summarized in Table 1 [1], The underlying mechanisms for APS remain unclear [3].

**Table 1:** Summary of the characteristics of different subtypes of APS. (Adapted from Husebye, Anderson, and Kämpe, 2018 [1]

	APS type 1	APS type 2	IPEX
Main Features	Addison’s disease Hypoparathyroidism Chronic mucocutaneous candidiasis	Addison’s disease Autoimmune thyroid disease Type 1 Diabetes mellitus	Autoimmune enteropathy Neonatal type 1 diabetes mellitus Eczema
Genetics	Monogenic (Autosomal recessive and dominant) AIRE gene	Polygenic	Monogenic (X-linked) FOXP3 gene
Onset	Childhood to adolescence	Adolescence to Adult	Infancy
Frequency	Rare (1:100,000)	Common (1:100)	Rare (1:1000,000)

The current case was an example of APS type 2 which may be characterized by at least two of three autoimmune conditions – Type 1 diabetes mellitus, autoimmune thyroid disease and Addison disease [4]. Similar to most autoimmune conditions, it occurs more commonly in females and may be associated with other autoimmune conditions such as celiac disease or autoimmune primary hypogonadism [1].

Given the number of organ systems that may be involved in this syndrome, APS type 2 can manifest in a myriad of ways, although pericarditis is a rare complication. Review of the literature reveals 13 affected patients who ranged in age from 21 to 54 years (mean 36.8 years) [3, 5–14]; there were no fatalities. Despite APS type 2 being more common in females, this particular manifestation seems to predominate in males with a ~ 2:1 ratio (8 males, 5 females), although the numbers are small.

In the reported cases treatment was undertaken with pericardiocentesis draining volumes of fluid ranging from 190-400mL [3, 5–14]. While these amounts may not necessarily cause hemodynamic instability [3], particularly if accumulation occurs slowly over time, the reported case had Addison disease with the likelihood of an impaired stress response [3]. In addition, a constrictive component has been described in pericardial efffusions in APS type 2 which may contribute to compromise of underlying myocardial function [13].

The reported patients all had autoimmune thyroid disease and adrenal insufficiency with two having evidence of autoimmune primary hypogonadism (both males) [7, 12]. None of the patients had T1DM although three (all male) had antibodies against glutamic acid decarboxylase-65 (GAD) [3, 8, 13]. One case had possible systemic lupus erythematosus (SLE) [10], and one had transglutaminase and endomysial antibodies, although with no biopsy-proven celiac disease [7].

In conclusion, a case of pericarditis with lethal cardiac tamponade secondary to APS type 2 is presented. The mechanism resulting in compromise of cardiac function involved space-occupying fluid within the pericardial sac impeding venous return to the heart and compressing cardiac chambers. A possible restrictive component may have been present against a background of Addison disease. Such cases should prompt careful evaluation of endocrine organs at autopsy.
